# Assessing the quality of service of village malaria workers to strengthen community-based malaria control in Cambodia

**DOI:** 10.1186/1475-2875-9-109

**Published:** 2010-04-23

**Authors:** Junko Yasuoka, Krishna C Poudel, Kalpana Poudel-Tandukar, Chea Nguon, Po Ly, Duong Socheat, Masamine Jimba

**Affiliations:** 1 Department of Community and Global Health, The University of Tokyo, 7-3-1 Hongo, Bunkyo-ku, Tokyo 113-0033, Japan; 2 Department of Epidemiology and International Health, National Center for Global Health and Medicine, 1-21-1 Toyama, Shinjuku-ku, Tokyo 162-8655, Japan; 3 National Centre for Parasitology, Entomology and Malaria Control, 372 Monivong Boulevard, Phnom Penh, Cambodia

## Abstract

**Background:**

Malaria continues to be a major public health problem in remote forested areas in Cambodia. As a national strategy to strengthen community-based malaria control, the Cambodian government has been running the Village Malaria Worker (VMW) project since 2001. This study sought to examine the nature and quality of the VMWs' services.

**Methods:**

Data collection was carried out in February and March 2008 through interviews with one of the two VMWs who takes the lead in malaria control activities in each of the 315 VMW villages (n = 251). The questionnaire addressed 1) the sociodemographic characteristics of VMWs, 2) service quality, 3) actions for malaria prevention and vector control, and 4) knowledge of malaria epidemiology and vector ecology.

**Results:**

VMWs were effective in conducting diagnosis with Rapid Diagnostic Tests (RDTs) and prescribing anti-malarials to those who had positive RDT results, skills that they had acquired through their training programmes. However, most other services, such as active detection, explanations about compliance, and follow-up of patients, were carried out by only a small proportion of VMWs. The variety of actions that VMWs took for malaria prevention and vector control was small (average action index score 12.8/23), and their knowledge was very limited with less than 20% of the VMWs giving correct answers to six out of seven questions on malaria epidemiology and vector ecology. Knowledge of vector breeding places and malaria transmission were significant determinants of both the quality of VMWs' services and the variety of their actions for malaria prevention and vector control.

**Conclusions:**

VMWs' services focused primarily on diagnosis and treatment. Their focus needs to be broadened to cover other aspects of malaria control in order to further strengthen community-based malaria control. VMWs' actions and knowledge also need substantial improvement. Strengthening training programmes can help achieve better performance by VMWs.

## Background

Malaria continues to be a major public health problem in Cambodia. The National Centre for Parasitology, Entomology and Malaria Control (CNM) reported 42,518 confirmed malaria cases in 2007, of which 87% were attributable to *Plasmodium falciparum *[1]. A recent large-scale malaria survey in Cambodia reported prevalence of 3.0%-12.3% in malaria-prone provinces [2]. Perennial malaria transmission is maintained in forest-covered hills and mountains, where the major vectors, such as *Anopheles dirus*, *Anopheles minimus*, and *Anopheles maculatus*, are widespread [3,4].

Despite continued efforts made by the CNM, there have been a number of obstacles to successful implementation of the malaria control programme. For example, malaria prevalence remains high in remote forested areas, which are difficult to access, especially in the rainy season [5]. It is hard to reach those who are at risk of malaria, especially migrants who recently moved into the remote forest, ethnic minorities living in thickly forested villages, and non-immune temporary forest workers [2,6]. Nearly 80% of patients with fever use private health services such as village vendors and private clinics, where government control is limited and the quality of diagnosis and treatment is often questionable [7,8]. It is very likely that widespread availability of counterfeit anti-malarials has been accelerating drug resistance in forested areas near the Thai-Cambodian border [9,10].

In order to increase access to accurate diagnosis and treatment in remote forested areas, the CNM launched the Village Malaria Worker (VMW) project in 2001. The CNM identified malaria-prone villages, where two VMWs (a male and a female) per village were selected through community consensus. During the last eight years, the project has been scaled up to 315 villages in seven remote provinces. The VMWs participated in a three-day training programme organized by the CNM, which covered malaria epidemiology, prevention, diagnosis using Rapid Diagnostic Tests (RDTs), treatment with artesunate and mefloquine (A+M), referral to hospitals, and recording of fever cases and positive RDT results. Different kinds of teaching materials were utilized in the training program, including RDT kits, blister-packed anti-malarials, bed nets, flipcharts, leaflets, and handouts with tables and figures. CNM also provided a refresher training programme. The curriculum contained practical demonstrations, such as recognizing the signs and symptoms of malaria, performing RDTs, prescribing correct dosages of anti-malarials, managing the VMW kit, and completing record forms. Trained VMWs are supposed to perform RDTs on any villager suspected of having malaria and, for test-positive cases, provide blister-packaged A+M according to the national guidelines. They are also encouraged to conduct active case detection, follow-up patients, and spread information on preventive measures to their villagers. VMWs are supervised and resupplied with RDT kits and anti-malarials monthly by the CNM [5,11]. The CNM directly supervise VMWs because they place high importance on the VMW project to promote community-based malaria control and because they aim at building strong working relationships with VMWs and obtaining timely information from the field.

Previous studies have demonstrated that VMWs are an effective means of improving access to early diagnosis and treatment of malaria in Cambodia. For example, the VMW project significantly increased the likelihood of villagers receiving a biological diagnosis and A+M [6,12]. Cost analysis of the VMW project showed that the cost per patient treated was $5.14 per falciparum malaria patient treated, which was found to be more cost-effective than other malaria outreach interventions [11].

However, scientific evaluation of the quality of the VMWs' services (service quality), their actions related to prevention, and their knowledge of malaria, has not been carried out in spite of the relatively long history of the project. Inadequate performance by community health workers is a widespread problem in many public health fields, including malaria [13-16]. Although a recent systematic review categorized and described several intervention models involving community health workers that aimed to improve case management at the community-level [17], few studies have conducted a detailed examination of the quality of malaria control activities by community health workers and determinants of their performance. Evaluation of community health worker performance, in general, is the focus of much attention at this time, as many countries invest in them as a strategy to reach the Millennium Development Goals [18,19]. A better understanding of the VMWs' services and their determinants is needed to evaluate and improve this project, and may contribute to improving community health worker performance in other areas as well.

This study, therefore, sought to: 1) examine the VMWs' service quality, their actions for malaria prevention and vector control, and their knowledge of malaria epidemiology and vector ecology; and 2) identify determinants of VMWs' service quality and actions for malaria control. Based on the results of this study, the importance of and strategies for improving community-based malaria control programmes will be discussed.

## Methods

### Study site

**Figure 1:**
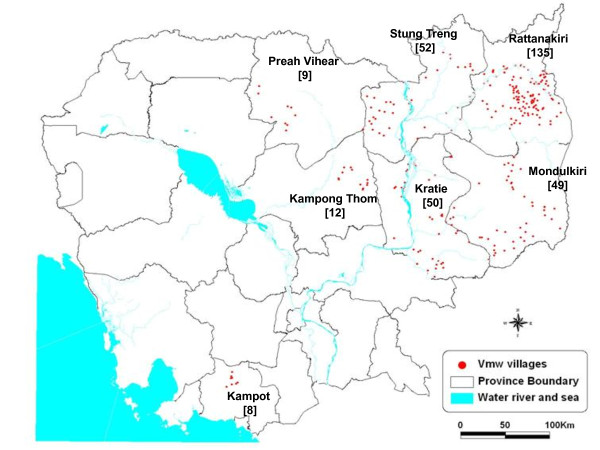
**Map of 315 VMW villages: name of province [number of VMW villages]**.

This cross-sectional study was conducted in seven remote provinces of Cambodia where the 315 VMW villages are located: Rattanakiri, Kratie, Mondurkiri, Stung Treng, Kampong Thom, Kampot, and Preah Vehear (Figure 1).

### Data collection

Data were collected from February 10 to March 8, 2008, through interviews with one of the two VMWs who takes the lead in malaria control activities in each of the 315 VMW villages (n = 251). Interviews were conducted in designated health centres in each province. Sixty-four VMWs were not able to participate in the survey due to their sudden illnesses, unexpected family obligations, and transportation problems.

The questionnaire addressed 1) the VMWs' sociodemographic characteristics, 2) service quality, 3) actions for malaria prevention and vector control, and 4) knowledge of malaria epidemiology and vector ecology. The questionnaire was developed in English, referring to a previous study's questionnaire [20], and translated into Khmer by a local malaria expert. It was then back-translated to English by another expert for confirmation. It was piloted by the authors with VMWs in Kampot province to determine if all items were understandable, if there were appropriate answers for each question, and to learn how comfortable VMWs felt taking the survey. Three CNM staff were hired and trained to conduct the survey, which was closely supervised by the authors.

### Measures

Two additive indices were developed to quantify the quality of VMWs' services and the variety of actions they took for malaria prevention and vector control (Table 1).

**Table 1:** Indices to measure VMWs' service quality and actions for malaria prevention and vector control

Index	Number of items in index	Maximum possible score	Mean	SD	Reliability (Chronbach's alpha)	Item
Service quality	5	5	3.174	0.920	0.828	Active detection
						Diagnosis and treatment
						Prescription of anti-malarials
						Follow-up
						Dissemination of preventive measures

Actions	2	23	12.833	4.400	0.828	Malaria preventive measures Vector control measures

The quality index was developed based on respondents' answers to questions regarding five items: active detection, diagnosis and treatment, prescription of anti-malarials, follow-up of patients, and dissemination of preventive measures. The action index was developed based on the variety of malaria preventive measures and vector control measures that the VMWs undertook themselves. The third topic, knowledge of malaria epidemiology and vector ecology, was measured by the respondents' correct answers on seven items related to those topics.

#### Quality index

Scores for active detection and follow-up were given according to the regularity or frequency of respondents' home visits to find malaria patients (regularly = 3, sometimes = 2, rarely = 1, never = 0) and to check if patients had recovered (always = 2, sometimes = 1, never = 0). Each service for diagnosis and treatment (conduct RDTs, observe symptoms, ask symptoms from family, take body temperature, and prescribe anti-malarials to those who had positive RDT results) was given a maximum of two points (always = 2, sometimes = 1, never = 0). Regarding prescription of anti-malarials, explanations about dosage and the importance of compliance were given a maximum of two points (always = 2, sometimes = 1, never = 0). Four items on how the VMWs explained about dosage and compliance were given one point each, if included. Dissemination of effective preventive measures was given a maximum of two points (always = 2, sometimes = 1, never = 0). Dissemination of less effective measures was given one point (always/sometimes = 1, never = 0), and of wrong measures, zero points (always/sometimes = 0, never = 1). The score for each of the five items was calculated as [total points divided by maximum points] so that each item is given a maximum of one point. These scores were added up to create the index (range: 0-5). The logic of combining these items was confirmed by a high Cronbach's alpha reliability score (0.828).

#### Action index

Each malaria preventive measure and vector control measure that the VMWs undertook themselves was given one or two points, according to its effectiveness and frequency. Effective measures were given a maximum of two points (always/most of the time = 2, sometimes/rarely = 1, never = 0). These measures included "come back home before dawn," "wear long-sleeved shirts/pants," "sleep under bed nets at home," "refrain from going to the forest," "bring hammock nets to the forest," "burn trash around house," "seal holes/cracks on walls/ceilings," and "cover water jars/tanks." Less effective measures were given a maximum of one point (always/most of the time/sometimes = 1, rarely/never = 0). These measures included "kill mosquitoes by hands," "use mosquito coils," "spray house," and "clear bush around house." A wrong measure, "plant flowers/grasses around house," was given 0 points (always/most of the time/sometimes/rarely = 0, never = 1). The total points became the index score, ranging from 0 to 23. The logic of combining these items was confirmed by a high Cronbach's alpha reliability score (0.828).

#### Knowledge

Knowledge of malaria epidemiology and vector ecology was measured based on the respondents' correct answers to questions regarding seven items: malaria symptoms, malaria transmission, vector species, vector active time, vector development time, breeding places, and natural enemies. Regarding malaria symptoms, respondents were asked if stomach ache, diarrhoea, nausea, fever, and shivering are the correct symptoms of malaria or not. Seven choices on malaria transmission routes were given: by cough or sneeze, touching blood, touching utensils, sharing food, coming close to mosquitoes, mosquito bites, and other. For vector species, choices on mosquito genera and sex were given to respondents. Regarding the vector's most active time, respondents were asked to choose one of four time periods, morning, afternoon, dusk to dawn (evening/night), and other. Vector development time was asked by an open-ended question. Choices given for vector breeding places were trees (branches/leaves), on the ground, water pools around houses, water pools in the forest, and other. For natural enemies of the vector, five choices were given, dogs, birds, aquatic insects, small fish, and other.

### Data management, statistical analysis, and ethical considerations

All survey data were coded, entered into data analysis software, and then double-checked by the authors to ensure accuracy. In order to identify determinants of the quality of VMWs' services and the variety of actions for malaria prevention and vector control (as represented by the quality index and action index), multiple linear regressions were run with sixteen independent variables: nine sociodemographic factors (age, education, gender, occupation, region, ethnicity, length of VMW career, most recent VMW training attended, and reasons for becoming VMWs) and the seven knowledge items described above. Data analysis was done using STATA version 9.

Informed consent was obtained from all participants before the interview. The project protocol, consent forms, and survey questionnaires were approved by the Ethical Committee of the University of Tokyo.

## Results

### Sociodemographic characteristics

From the 315 VMW villages, 251 VMWs took part in the survey. Their sociodemographic characteristics are described in Table 2. Respondents' ages ranged from 15 to 70 years (mean 35.4) and school attendance from 0 to 12 years (mean 3.7). Male participation was higher (80.9%) than female, which indicates that male VMWs played a central role in VMW activities in the majority of VMW villages. Respondents have been serving as VMWs for about 3.3 years (mean), and the majority of them (85.7%) attended VMW training (for new VMWs) or refresher training between 1 and 1.5 years ago. About half of them (51.8%) became VMWs because they were recommended by villagers, and the other half (48.2%) did so because they were interested in malaria control.

**Table 2:** Selected sociodemographic characteristics of the study population

Characteristics (n = 251)	Mean	SD	Number	%Total
Age	35.4	12.3		
Education (final grade)	3.7	2.4		
Gender				
Male			203	80.9
Female			48	19.1
Occupation				
Farmer			237	94.4
Other			14	5.6
Region				
Mountainous			148	59.0
Other			103	41.0
Ethnicity				
Khmer			87	34.7
Other			164	65.3
VMW career (months)	40.2	14.2		
Most recent VMW training attended (months ago)	16.0	5.0		
Reason for becoming VMW				
Recommended by villagers			130	51.8
Interested in malaria treatment/prevention			121	48.2

### Service quality

The survey revealed a substantial gap in the VMWs' level of achievement on the various service items (Table 3). In general, VMWs were very effective in conducting what they had learned in VMW training programme. Almost all VMWs were able to perform RDTs and prescribe blister-packed A+M to those who had positive RDT results (99.2% and 97.6%, respectively). When prescribing anti-malarials, 99.2% of the respondents explained about dosage to their patients.

**Table 3:** Responses from the study population (n = 251)

		n	%
**Service quality**			
Active detection	Visit villagers to find malaria patients (Regularly)	68	27.1
Diagnosis and treatment	Use RDTs (Always)	249	99.2
	Observe symptoms (Always)	119	47.4
	Ask symptoms from family (Always)	66	26.3
	Take body temperature (Always)	61	24.3
	Prescribe A+M to those who had positive RDT results (Always)	245	97.6
Prescription of anti-malarials	Explain about dosage (Always)	249	99.2
	Explain about the importance of compliance (Always)	140	55.8
	Compliance failure can result in incomplete treatment	182	72.5
	Inappropriate to save tablets to treat other people's malaria	174	69.3
	Inappropriate to save tablets for next infection	166	66.1
	Compliance failure can cause/spread drug resistance	16	6.4
Follow-up	Make home visits or ask patients' family to check if patients recovered (Always)	48	19.1
Dissemination of preventive measures	Sleep under bed nets (Always)	198	78.9
	Bring hammock nets to forest (Always)	145	57.8
	Clear bush around house (Always)	91	36.3
	Fill in water pools (Always)	70	27.9
	Wear long-sleeve shirts/pants (Always)	58	23.1
	Should not come close to malaria patients (Always/Sometimes)	53	21.1
	Should not share utensils with malaria patients (Always/Sometimes)	42	16.7
	Cover water jars/tanks (Always)	36	14.3
	Spray house (Sometimes)	24	9.6
	Use mosquito coils (Always)	1	0.4
**Actions for malaria prevention and vector control**			
Malaria preventive measures (Always/Most of the time)	Sleep under bed nets at home	227	90.4
	Bring hammock nets to the forest	138	55.0
	Wear long-sleeved shirts/pants	121	48.2
	Come back home before dawn	117	46.6
	Refrain from going to the forest	116	46.2
Vector control measures (Always/Most of the time)	Burn trash around house	117	46.6
	Clear bush around house	102	40.7
	Fill in water pools	81	32.3
	Kill mosquitoes by hands	80	31.9
	Cover water jars/tanks	65	25.9
	Seal holes/cracks on walls/ceilings	6	2.4
	Plant flowers/grasses around house	5	2.0
	Spray house	2	0.8
	Use mosquito coils	2	0.8
**Knowledge of malaria epidemiology and vector ecology **(VMWs who gave correct answers)			
Malaria symptoms		28	11.2
Malaria transmission		49	19.5
Vector species		25	10.0
Vector active time		246	98.0
Vector development time		17	6.8
Vector breeding places		14	5.6
Natural enemies of vector		4	1.6

However, respondents' performance was inadequate related to service items that were not well covered in the training programme. For example, only about one-quarter of VMWs (27.1%) regularly conducted active detection of malaria patients. When diagnosing malaria, less than half of them (47.4%) took symptoms into consideration. When providing anti-malarials, only about one-half of them (55.8%) explained about the importance of compliance. Related to the contents of their explanations, the majority explained that compliance failure can result in incomplete treatment (72.5%) and that it is inappropriate to save tablets for other people or for a future infection (69.3%). However, only 6.4% explained that non-compliance can spread drug resistance. Only 19.1% of the respondents followed up with patients to make sure that they had recovered from malaria. Regarding their dissemination of information on preventive measures, the majority recommended bed net use at home (78.9%) and/or in the forest (57.8%). However, 21.1% told villagers not to come close to malaria patients and 16.7% told them not to share utensils with patients, which indicates considerable misunderstanding of the route of malaria transmission.

### Actions for malaria prevention and vector control

VMWs' actions to protect themselves from malaria and to reduce the burden of mosquito vectors were found to be limited. Although bed net use was practiced at home by most of the respondents (90.4%), other self-protection measures were taken by only half of them (46.2%-55.0%). Regarding vector control, "burn trash around house" and "clear bush around house" were the most common measures taken (46.6% and 40.7%, respectively), but none of the measures were taken by more than half of them (0.8%-46.6%).

### Knowledge of malaria epidemiology and vector ecology

The survey results clearly demonstrated VMWs' insufficient knowledge of malaria epidemiology and vector ecology. It was striking that less than 20% of them were able to give correct answers to questions about six topics: malaria symptoms, transmission, vector species, development time, breeding places, and natural enemies. The only topic well known by most of the VMWs (98.0%) was the vector active time, which can be answered based on their observations and experience, rather than by education. There was little variation in knowledge level among the VMWs, and the level was quite low as a whole.

### Determinants of service quality and action

With the survey data, multiple linear regression analysis was run to identify determinants of the quality of VMWs' services (Table 4). Significant determinants of the quality of VMWs' services were occupation (Beta = 0.558, p = 0.003), length of VMW career (Beta = 0.007, p = 0.023), reason for becoming a VMW (Beta = -0.649, p < 0.001), knowledge of malaria transmission (Beta = 0.989, p < 0.001), knowledge of vector species (Beta = 0.307, p = 0.042), and knowledge of vector breeding places (Beta = 0.574, p < 0.001). As a result, being a farmer was positively related to better service quality, compared to having other occupations, such as shop keeper, teacher, or forest worker. A longer VMW career was associated with better service quality. VMWs who said that they were recommended by villagers, as the main reason for becoming VMWs, were found to provide better quality services than those who became VMWs mainly because of their interests in malaria treatment or prevention. More knowledge of malaria transmission, vector species, and vector breeding places was associated with better service quality.

**Table 4:** Determinants of VMWs' service quality

	Beta coefficient	SE	t	p-value
Occupation	0.558	0.187	2.98	0.003
Length of VMW career	0.007	0.003	2.28	0.023
Reason for becoming VMW	-0.649	0.089	-7.33	<0.001
Knowledge of malaria transmission	0.989	0.136	7.30	<0.001
Knowledge of vector species	0.307	0.151	2.04	0.042
Knowledge of vector breeding places	0.574	0.139	4.13	<0.001

(Adjusted R^2 ^= 0.4815 for the best model by backward elimination)

The other regression model revealed that significant determinants of the variety of VMWs' actions for malaria prevention and vector control included occupation (Beta = 5.634, p < 0.001), most recent VMW training attended (Beta = -0.168, p < 0.001), reason for becoming a VMW (Beta = -1.966, p < 0.001), knowledge of malaria transmission (Beta = 2.185, p = 0.002), and knowledge of vector breeding places (Beta = 3.749, p < 0.001) (Table 5). As a result, being a farmer was positively related to the variety of actions, compared to having other occupations. Attending VMW trainings (including refresher trainings) more recently had a positive impact on the variety of actions. VMWs who said that they were recommended by villagers, as the main reason for becoming VMWs, were found to conduct more variety of actions than those who became VMWs mainly because of their interests in malaria treatment or prevention. More knowledge of malaria transmission and vector breeding places was associated with more variety of actions.

**Table 5:** Determinants of the variety of VMWs' actions for malaria prevention and vector control

	Beta coefficient	SE	t	p-value
Occupation	5.634	0.961	5.86	<0.001
Most recent VMW training attended	-0.168	0.044	-3.79	<0.001
Reason for becoming VMW	-1.966	0.458	-4.30	<0.001
Knowledge of malaria transmission	2.185	0.706	3.09	0.002
Knowledge of vector breeding places	3.749	0.717	5.23	<0.001

(Adjusted R^2 ^= 0.3948 for the best model by backward elimination)

From the two regression models described above, occupation, reason for becoming a VMW, knowledge of malaria transmission, and knowledge of vector breeding places were found to be important determinants for both the quality of VMWs' services and VMWs' actions for malaria prevention and vector control.

## Discussion

This study revealed that almost all VMWs were able to conduct diagnosis with RDTs and prescribe anti-malarials to those who had positive RDT results, skills that they had acquired through the VMW training programmes organized by the CNM. However, other service items were not performed well, and VMWs' actions for malaria prevention and vector control and knowledge of malaria epidemiology and vector ecology were limited. In fact, active detection, explanations about compliance, and follow-up of patients were carried out by only a small proportion of VMWs. Dissemination of preventive measures focused primarily on bed net use, and about 20% of the VMWs spread wrong information about the transmission route. The variety of actions that VMWs took for malaria prevention and vector control was small, and their knowledge was extremely limited, with less than 20% of the VMWs giving correct answers to six out of seven questions on malaria epidemiology and vector ecology.

Several studies have described weaknesses in the community health worker approach in low- and middle-income countries. For example, one review concluded that community health workers did not consistently provide services that were likely to have substantial effects on health and that quality was usually poor [18,21]. A recent study reported poor performance of Village Health Workers in Viet Nam, a neighbouring country of Cambodia. It suggested that weaknesses in their malaria management were attributable to several underlying influences, including insufficient time to complete duties outside of normal working hours, inadequacies in pre- and in-service training, and some delays in rolling out the new guidelines for drugs in Village Health Worker kits [14].

A number of studies have discussed different kinds of strategies for improving health-worker performance. A recent review reported that the simple dissemination of written guidelines is usually ineffective, supervision and audit with feedback is generally quite effective, and multifaceted approaches (e.g., training plus supervision) may be more effective at changing practices than single-component interventions [13]. The importance of supervision is particularly emphasized because it can improve performance at least in the short-term [22], provide professional development, and improve health workers' job satisfaction and motivation [23].

One possible strategy to expand the range of VMWs' services and to improve their actions and knowledge would be to strengthen the VMW training curriculum. Since most of the VMWs were able to accomplish what they had learned in VMW trainings, there is a good possibility that improved training programmes could achieve wider service range and higher service quality. Although VMWs' services have been focused on diagnosis and treatment so far, they should be expanded to other aspects of malaria control, including prevention and vector control. In fact, regression analyses of this study demonstrated that knowledge of malaria transmission and that of vector ecology, especially regarding vector breeding places, are significant determinants for both VMWs' service quality and actions for malaria control. This is possibly because overall understanding of malaria control, including both malaria epidemiology and vector ecology, is necessary to provide better service quality and more actions. In order to further promote community-based malaria control in Cambodia, the range of VMWs' services should be widened to cover more aspects of malaria control, and VMWs should take the initiative in conducting a greater variety of measures for malaria prevention and vector control.

Specifically, the current VMW training curriculum can be improved by: 1) making its contents easier to understand for VMWs with limited education (three years of school education on average), especially technical terms, tables, and figures in flipcharts, leaflets, and handouts, 2) including a variety of topics regarding malaria and vector control, and 3) adding more participatory activities to provide hands on experience. Although there is a need to break away from an old paradigm that performance can be improved by training alone [13], some modifications in the curriculum and inclusion of more participatory activities in the training could lead to the acquisition of accurate knowledge and skills among VMWs in the long run. Further research is now underway to examine the effectiveness of a new training programme developed based on the results of this study in achieving higher VMW service quality.

As described in previous studies [13,22,23], strengthening supervision in combination with modifying the training curriculum might be effective in improving VMWs' service quality. Currently, supervision is conducted by the CNM staff in two ways: monthly meetings at health centers in each region and village visits twice a year. In monthly meetings, CNM staff check VMWs' records of fever cases and RDT positive cases, and resupply RDT kits and anti-malarials. They also visit each VMW village twice a year to directly monitor VMW activities and observe their relationship and communication with villagers. In addition to these components of current supervision, providing VMWs with opportunities to review their knowledge of malaria control (especially prevention) and to share their experiences and challenges could be an effective means to improve their knowledge and motivation. Also, increasing the frequency of village visits could facilitate professional development and improve the two-way flow of information between VMWs and the central government.

Some limitations of this research must be taken into account when interpreting study findings. To evaluate VMWs' service quality, only self-reported data were used, and actual villagers' experiences were not taken into account. This limitation can partly be covered by another recent study, which examined VMWs' service quality using data obtained from villagers. The validation of self-reported indices regarding service quality and action needs improvement. However, possible attempts were made: for example, self-reported data were double-checked with VMWs' records in their monthly reports, which are submitted to the CNM regularly. In addition, objective information about VMWs' service quality was obtained from local health workers as much as possible.

Several Asian and African countries are currently investing in community health workers as a major part of their strategies to reach the Millennium Development Goals [18]. For example, nearly 25,000 community health workers have been trained in Ethiopia and are delivering family planning, immunization, and health education to their communities [24]. In India, state-wide community health worker programmes are under way as a part of the National Rural Health Mission. Furthermore, several trials have been carried out to expand community health workers' roles. For example, the role of 54,000 women community volunteers in India evolved over time into two different sets of activities: one focuses on child survival and the other on women's empowerment and community action [25]. Also, a trial to expand Cambodian VMWs' role to the management of diarrhoea and acute respiratory diseases started in 2005 [11]. Since there is substantial interest in the potential contributions of community health workers to reach the Millennium Development Goals, it is timely and vital to examine their performance and propose effective strategies to support them in their work and to improve the quality of their services in different settings.

## Conclusions

This study has demonstrated that Cambodian VMWs' services focused narrowly on diagnosis and treatment, and that their knowledge of malaria epidemiology and vector ecology as well as actions for malaria prevention and vector control require substantial improvement. Knowledge of malaria transmission and that of vector breeding places were found to be significant determinants of both VMWs' service quality and actions for malaria prevention and vector control. In addition to diagnosis and treatment, which have been the focus of VMWs' services so far, more aspects of malaria control should be covered in their training to further promote community-based malaria control in the country.

## Competing interests

The authors declare that they have no competing interests.

## Authors' contributions

JY conceived the study, developed the questionnaire, analysed data, and wrote the manuscript. KCP contributed to the study design, trained surveyors, conducted fieldwork, and improved the manuscript. KPT entered and analysed data and improved the manuscript. CN, PL, and DS supervised fieldwork. MJ monitored the study progress and provided guidance to improve the manuscript. All authors read and approved the final draft.
